# An *Acinetobacter baumannii* nasal carriage isolate recovered from an asymptomatic patient in Vietnam is extensively antibiotic resistant and produces a rare K71 type capsule

**DOI:** 10.1128/spectrum.01838-24

**Published:** 2024-10-22

**Authors:** Anna M. Shpirt, Christopher J. Harmer, Alexander S. Shashkov, Mikhail M. Shneider, Alexander O. Chizhov, Andrey S. Dmitrenok, Anastasiya V. Popova, Anastasiya A. Kasimova, Andrei V. Perepelov, Yuriy A. Knirel, Ruth M. Hall, Johanna J. Kenyon

**Affiliations:** 1 N. D. Zelinsky Institute of Organic Chemistry, Russian Academy of Sciences, Moscow, Russia; 2 School of Life and Environmental Sciences, The University of Sydney, Sydney, Australia; 3 M. M. Shemyakin & Y. A. Ovchinnikov Institute of Bioorganic Chemistry, Russian Academy of Sciences, Moscow, Russia; 4 State Research Center for Applied Microbiology and Biotechnology, Obolensk, Moscow Region, Russia; 5 School of Pharmacy and Medical Sciences, Health Group, Griffith University, Gold Coast Campus, Southport, Queensland, Australia; Centre de Biologie Integrative, Toulouse, France

**Keywords:** *Acinetobacter baumannii*, capsular polysaccharide, K locus, K71, rhamnose

## Abstract

*Acinetobacter baumannii* is a leading cause of multidrug-resistant bacterial infections worldwide, and the capsular polysaccharide (CPS) is a major virulence determinant. A previous study of *A. baumannii* from intubated and asymptomatic patients admitted to the intensive care unit (ICU) at the Hospital for Tropical Diseases in Ho Chi Minh City in Vietnam revealed multiple lineages with diverse antibiotic resistance profiles and CPS biosynthesis loci. Here, we show that 48_n, an asymptomatic nasal carriage isolate belonging to ST142, is extensively antibiotic resistant and carries acquired resistance determinants accounting for the resistance profile. 48_n carries the novel KL71 CPS biosynthesis locus in the chromosome. The structure of the CPS produced by 48_n was established using ^1^H and ^13^C nuclear magnetic resonance spectroscopy, including two-dimensional ^1^Н,^1^Н COSY, ^1^Н,^1^Н TOCSY, ^1^Н,^1^Н ROESY, ^1^Н,^13^C HSQC, and ^1^Н,^13^C HMBC experiments, and confirmed by Smith degradation. Consistent with the genetic content of KL71, the K71 CPS was found to be made up of octasaccharide K units containing six l-rhamnose residues and one residue each of *N*-acetyl-d-glucosamine and d-glucuronic acid. K71 CPS was branched and closely related to the K74 CPS produced by BAL_309, an antibiotic susceptible ST142 isolate recovered from an intubated patient in the same ICU 7 years later. K71 and K74 differ only in the linkage between K units, and this is due to the replacement of a single gene at the K locus that codes for the Wzy polymerase.

**IMPORTANCE:**

The majority of *Acinetobacter baumannii* genomes sequenced and analyzed to develop an understanding of extensively drug-resistant (XDR) isolates belong to the globally disseminated CC2 clonal complex. While XDR isolates belonging to rarer lineages are often unexplored, detailed analyses could provide novel insights into the spread of resistance, as well as cell surface features such as the CPS that determine the specificity of non-antibiotic therapeutics required to treat XDR infections that resist antimicrobial chemotherapy. Here, we describe the properties of an XDR asymptomatic nasal carriage isolate recovered in Vietnam that belongs to ST142, a rarely encountered sequence type. We report the resistance profile and correlate this with detected resistance determinants. We also solve the structure of the CPS and reveal its relationship with CPS produced by other *A. baumannii* isolates.

## INTRODUCTION

*Acinetobacter baumannii* is an opportunistic Gram-negative bacterial pathogen that is listed among the leading etiological agents of mortality attributed to multidrug resistance worldwide (1). Alternate therapeutic interventions being investigated for clinical use include monoclonal antibodies (2) and bacteriophage (3) that target the capsular polysaccharide (CPS) on the cell surface. The specific structure of the CPS produced by individual isolates can vary greatly in carbohydrate composition, the number and types of glycosidic linkages, and non-carbohydrate additions. Hence, elucidation of the structures of CPS produced by multiple, extensive, and pan antibiotic-resistant isolates is important to provide an understanding of target specificity that underpins the design and development of these therapies.

Previously, a study reported the short read data for 159 antibiotic-resistant *A. baumannii* isolates recovered from patients admitted to the intensive care unit (ICU) at the Hospital for Tropical Diseases (HTD) in Ho Chi Minh City, Vietnam (4). These isolates were obtained from specimens taken from patients assessed for asymptomatic colonization (carriage) following admission to the ICU during 2003–2007, or from intubated patients with ventilator-associated pneumonia (VAP) recovered during an outbreak that occurred between 2008 and 2012. Among this collection, several strains harbored novel CPS loci, and we have since reported the CPS structures for four of them (5–7).

While most isolates belonged to the major globally disseminated clonal complex, CC2 (also known as global or international clone 2), two were reported to belong to sequence type (ST) 142 and carry different sequences at the CPS biosynthesis K locus (KL). One of these isolates, BAL_309, was recovered in 2011 from a VAP patient during the ICU outbreak and was reported to be susceptible to antibiotics and carry the KL74 CPS locus (4). We previously determined the structure of the K74 CPS produced by BAL_309 and showed that it was composed of octasaccharide units consisting of l-rhamnose (l-Rha*p*), d-glucuronic acid (d-Glc*p*A), and *N*-acetyl-d-glucosamine (d-Glc*p*NAc) (6).

The second ST142 isolate, 48_n, was recovered in 2004, 7 years earlier than BAL_309, from a patient assessed for asymptomatic nasal carriage and was reported to carry a different set of genes, designated KL71, at the CPS biosynthesis locus (4). It was recorded as being resistant to amikacin, ceftazidime, ceftriaxone, and cefalexin, as well as having intermediate resistance to piperacillin/tazobactam, but sensitive to imipenem, ciprofloxacin, and colistin. In this study, we report an extended resistance profile and the genes found in the 48_n genome that account for its antibiotic resistance profile. We also describe the gene content of KL71 and its relationship to KL74. We determined the structure of the K71 CPS produced by 48_n and correlate this to the genetic content of the KL71 locus. We also describe the relationship between isolates 48_n and BAL_309.

## RESULTS

### *A. baumannii* 48_n is an extensively antibiotic-resistant isolate

The 48_n isolate was recorded as resistant to beta-lactams ceftriaxone and ceftazidime but susceptible to imipenem. It was also resistant to aminoglycosides amikacin and gentamicin and susceptible to ciprofloxacin and colistin (4). Using a more extensive group of antibiotics, here, resistance to the carbapenems meropenem and doripenem was detected as well as resistance to additional aminoglycosides, tobramycin and netilmicin, kanamycin and neomycin, and streptomycin and spectinomycin. 48_n was also resistant to sulfonamides, tetracycline, doxycycline, and trimethoprim but susceptible to fluoroquinolones. Hence, this sporadic carriage isolate was extensively antibiotic resistant.

The draft genome sequence of *A. baumannii* 48_n was assembled from the available short read data [Sequence Read Archive (SRA) accession number ERR197591] and was submitted to National Center for Biotechnology Information (NCBI) under accession number JAWXYJ000000000 (BioProject number PRJNA893697 and BioSample number SAMN38236054). The genome was found to belong to ST142^IP^ in the Institut Pasteur (IP) scheme and ST3245^OX^ in the Oxford (OX) scheme and to carry the *oxaAb* gene variant encoding OXA-510. Genes accounting for the resistance profile were detected; aminoglycoside resistance genes *aacC2d*, *aadB*, *aphA6*, and *aadA1* account for the observed aminoglycoside resistance pattern, *sul1* and *sul2* for sulfonamide resistance, *tet(X3*) for tetracycline resistance, and *dfrA41* for trimethoprim resistance. Three genes encoding beta-lactamases were present (*oxa10*, *bla*_CARB-49_, and *bla*_VEB-7_), and the *oxa10* gene likely accounts for the observed carbapenem resistance (8). The *cmlA1*, *floR*, and *arr2* genes and the *msrE-mphE* gene pair were also detected. The array of cassette-associated genes, *aadB:arr2:cmlA1:oxa10:aadA1*, was found together with *sul1* on a single contig, but the location of these and other resistance genes could not be determined from the draft genome. Some may be on the r3-T13 and r3-T21 plasmids detected.

### The KL71 CPS biosynthesis locus

**Fig 1:**
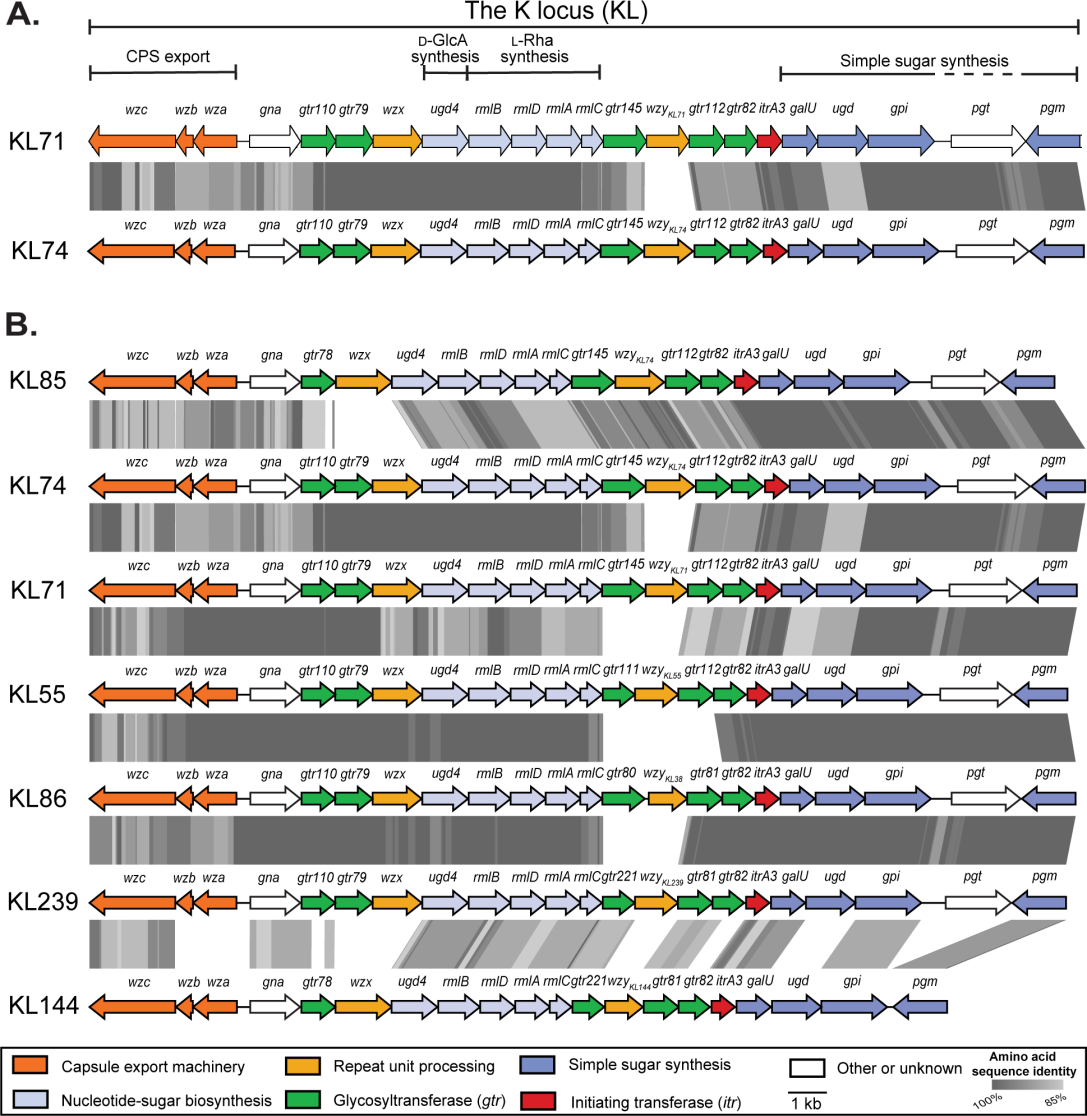
Organization of the KL71 locus and related K loci. Genes drawn as arrows are colored according to predicted functions of gene products as indicated by the scheme below. Gray shading indicates regions of >85% amino acid sequence identity between predicted proteins determined by tBLASTx using EasyFig v*.2.2.2* (13). (**A**) Comparison of KL71 with KL74. (**B**) Comparison of KL sequences from isolates producing CPS related to K71. Figure annotated in Adobe Illustrator. Figure drawn to scale using sequences available in the *A. baumannii* KL reference sequence database (14).

The sequence at the K locus in the 48_n genome was previously identified as KL71 (4), though the genetic content and arrangement of KL71 was not described. KL71 (Fig. 1A, annotated sequence released under GenBank accession number PP621479.1) includes a module of CPS export genes (*wza-wzb-wzc*) and a module of genes for simple nucleotide-linked sugars and precursors (*galU-pgm*) (9) on either side of a central region that includes *rmlBDAC* genes for synthesis of l-Rha*p* (10), a *ugd4* gene for conversion of UDP-d-Glc*p* to UDP-d-Glc*p*A (6, 11, 12), five glycosyltransferase genes (*gtr#*), a *wzx* translocase gene, and a *wzy* polymerase gene. It also includes a gene for the ItrA3 initiating transferase that begins K-unit synthesis on the inner membrane via transfer of d-Glc*p*NAc-1P to the undecaprenol phosphate lipid carrier (10).

A pairwise sequence comparison of KL71 and the KL74 sequence in the BAL_309 chromosome (GenBank accession number MN148383.1) revealed that the two sequences share 96.97% nucleotide sequence identity over 95% of the locus. KL71 and KL74 (Fig. 1A) share 21 of 22 genes, differing only in the sequence of the gene for the Wzy polymerase responsible for polymerizing K units to form the long-chain CPS. In the K74 CPS, Wzy_KL74_ forms a β-d-Glc*p*NAc-(1→3)-d-Glc*p*A linkage between octasaccharide units (6). Although Wzy_KL71_ and Wzy_KL74_ polymerases (GenPept accession numbers WZD86369 and QHE90354.1, respectively) both belong to the EpsG protein family (IPR049458/PF14897), the translated protein sequences do not share significant sequence identity. Hence, KL71 is predicted to produce the same octasaccharide K unit as KL74, but the K71 and K74 CPS structures are likely to have a different linkage between K units.

### Structure of the K71 CPS produced by *A. baumannii* 48_n

A CPS sample was isolated from cells of *A. baumannii* 48_n by phenol–water extraction (15). Sugar analysis of the CPS revealed rhamnose (Rha) and glucosamine in the ratio 2.9:1.0 (GLC detector response). The l configuration of rhamnose was established by GLC of the acetylated (*S*)−2-octyl rhamnosides (16). The d configuration of GlcNAc was established by the ^13^C nuclear magnetic resonance (NMR) data of the CPS using known regularities in glycosylation effects (17).

**Fig 2:**
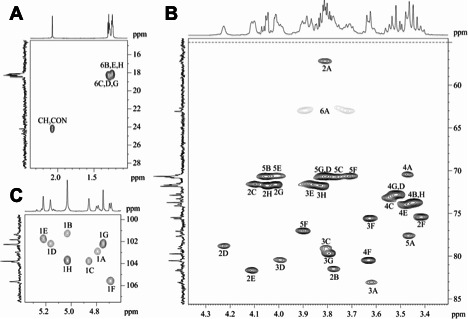
Expansion of ^1^H,^13^C HSQC spectrum of K71 OPS. (**A)** Methyl region. (**B)** Ring region. (**C)** Anomeric region. Arabic numerals refer to the carbon atoms in the residues as designated in Table 1.

In the ^1^H and ^13^C NMR spectra and a two-dimensional ^1^Н,^13^C HSQC spectrum (Fig. 2) of the CPS, there were eight signals for anomeric atoms, including those for six deoxyhexose (Rha*p*) residues. The presence of signals for an NAc group at δ_H_ 2.07 in the ^1^H NMR spectrum and δ_C_ 24.0 (Me) and 175.6 (CO) in the ^13^C NMR spectrum as well as a signal at δ_C_ 56.9 for C-2 in the ^13^C NMR spectrum showed that one Glc*p*NAc residue was present. A correlation between a CO_2_H group (δ_C_ 175.1) and H-5 at δ_H_ 3.90 in the two-dimensional ^1^H,^13^С HMBC spectrum showed that a glucuronic acid (Glc*p*A) residue was present. Therefore, the CPS has an octasaccharide repeating unit containing six Rha*p* residues and one residue each of Glc*p*NAc and Glc*p*A.

The ^1^Н and ^13^С NMR spectra of the CPS were assigned using two-dimensional ^1^Н,^1^Н COSY, ^1^Н,^1^Н TOCSY, ^1^Н,^1^Н ROESY, and ^1^Н,^13^C HSQC experiments (Table 1). The configurations of the glycosidic linkages were established by ^13^C NMR chemical shifts of C-5 compared with published data of the corresponding α- and β-pyranosides (18, 19). The β configuration of the Glc*p*NAc (**A**) and Glc*p*A (**F**) residues was confirmed by relatively large coupling constants *J*_1,2_ 8.5 and 7.8 Hz, respectively, and by H-1–H-3 and H-1–H-5 correlations in the ^1^H,^1^H ROESY spectrum of the CPS. The α configuration of the Rha residues (**B**, **C**, **D**, **E**, **G**, and **H**) was confirmed by H-1–H-2 correlations in the ^1^H,^1^H ROESY spectrum with no H-1–H-3 and H-1–H-5 correlations.

**TABLE 1:** Chemical shifts in the ^1^H and ^13^C NMR spectra (δ, ppm)^*a*^*^,^*^*b*^

Monosaccharide residue^*c*^		C1	C2	C3	C4	C5	C6
		*H1*	*H2*	*H3*	*H4*	*H5*	*H6 (6a,6b)*
CPS							
→3)-β-d-Glc*p*NAc-(1→	A	102.7 *4.79*	56.9 *3.81*	82.9 *3.62*	70.2 *3.47*	77.4 *3.47*	62.8 *3.71, 3.89*
→2)-α-l-Rha*p*-(1→	B	101.1 *5.03*	81.3 *3.78*	71.5 *3.85*	73.5 *3.46*	70.4 *3.78*	18.1 *1.28*
→3)-α-l-Rha*p*-(1→	C	103.6 *4.86*	71.4 *4.11*	78.9 *3.81*	73.1 *3.55*	4.05 *70.4*	17.9 *1.25*
→2,3)-α-l-Rha*p*-(1→	D	102.0 *5.17*	78.6 *4.23*	80.3 *4.00*	72.6 *3.52*	70.4 *3.82*	18.1 *1.30*
→2)-α-l-Rha*p*-(1→	E	101.6 *5.22*	81.4 *4.11*	71.4 *3.86*	73.4 *3.48*	70.4 *3.79*	18.1 *1.29*
→4)-β-d-Glc*p*A-(1→	F	105.4 *4.69*	75.2 *3.42*	75.4 *3.63*	80.3 *3.63*	76.8 *3.90*	175.1
→3)-α-l-Rha*p*-(1→	G	102.0 *4.75*	71.4 *4.02*	79.5 *3.80*	72.6 *3.52*	70.4 *4.00*	17.9 *1.24*
α-l-Rha*p*-(1→	H	103.6 *5.09*	71.4 *4.05*	71.7 *3.82*	73.9 *3.46*	70.4 *3.72*	17.9 *1.25*
OS^*d*^							
β-d-Glc*p*NAc-(1→	**A**'^*e*^	104.1 *4.74*	57.5 *3.75*	75.3 *3.57*	71.5 *3.46*	77.3 *3.45*	62.3 *3.76, 3.92*
→3)-α-l-Rha*p*-(1→	**D**'	103.4 *5.04*	71.4 *4.27*	75.3 *3.57*	73.0 *3.55*	70.7 *3.96*	18.2 *1.28*
→3)-α-l-Rha*p*-(1→	**C**'	101.0 *4.97*	71.8 *4.08*	80.0 *3.87*	72.6 *3.52*	70.8 *3.83*	18.2 *1.28*
→2)-R	**B**'	90.9 *5.11*	81.6 *3.64*	61.2 *3.74; 3.86*			

^*a*^ Structures of the K71 CPS and OS are shown in Fig. 3. ^1^H NMR chemical shifts are shown in italics.

^*b*^ R indicates glyceraldehyde in the hydrated form HOCH_2_CH(O–)CH(OH)_2_.

^*c*^ Signals for the N-acetyl group of Glc*p*NAc are at δ_С_ 24.0 (Me) and 175.6 (CO), δ_H_ 2.07 in the CPS, δ_С_ 23.8 (Me) and 176.2 ppm (CO), δ_H_ 2.04 in the OS.

^*d*^ OS, oligosaccharide.

^*e*^ Bold face text refers to the residues detected and their order

**Fig 3:**
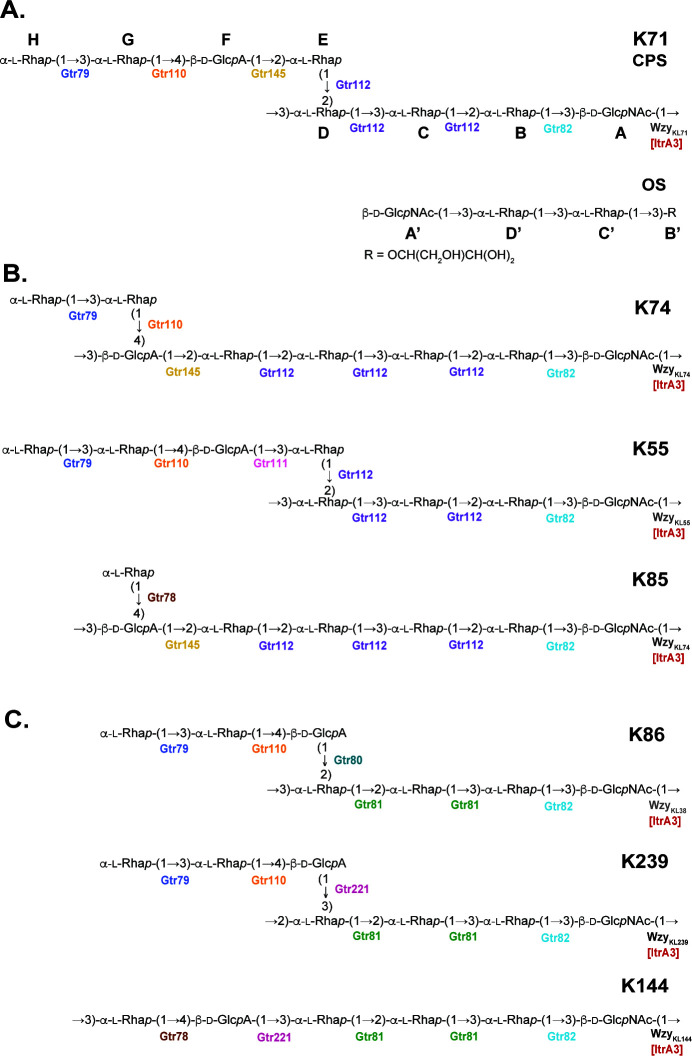
(**A**) Structures of the K71 CPS and OS. (**B** and **C**) Structures of *A. baumannii* CPS types related to K71. Individual glycosyltransferases (Gtrs), Wzy polymerases, and initiating transferases (Itrs) are colored and indicated next to the linkages they are predicted to form.

The glycosylation pattern of the monosaccharides was established by low-field positions of the linkage carbons: C-3 of units **A**, **C**, and **G**, C-2 of units **B** and **E**, C-2 and C-3 of unit **D**, and C-4 of unit **F** at δ 78.6–82.9 in the ^13^C NMR spectrum of the CPS, as compared with their positions at δ 71–75 in the corresponding non-substituted monosaccharides (18, 19). The observed downfield shifts were due to glycosylation effects on the linkage carbons (18).

**Fig 4:**
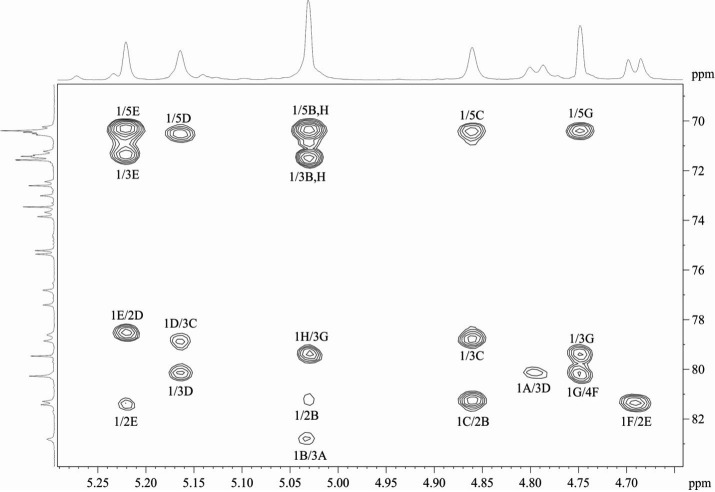
Anomeric region of a ^1^H,^13^C of HMBC of the K71 CPS. Arabic numerals before slash refer to protons and after slash to carbons of sugar residues as designated in Table 1.

**Fig 5:**
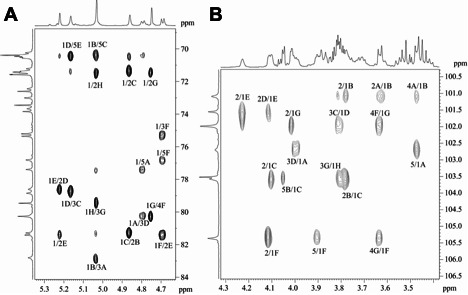
Expansion of ^1^H, ^13^C HSQC–NOESY spectrum of K71 OPS. (**A)** Anomeric region. (**B)** Ring region. Arabic numerals before slash refer to the protons, and those after slash refer to carbon atoms in the corresponding residues.

Linkage and sequence analyses of the CPS were performed using a two-dimensional ^1^H,^13^C HMBC experiment (Fig. 4). The HMBC spectrum showed *inter alia* the following inter-residue ^1^H/^13^C correlation peaks **A** H-1/**D** C-**3**, **B** H-1/**A** C-**3**, **C** H-1/**B** C-**2**, **D** H-1/**C** C-3, **E** H-1/**D** C-2, **F** H-1/**E** C-2, **G** H-1/**F** C-4, and **H** H-1/**G** C-3. Accordingly, the following inter-residue correlations between the anomeric protons and linkage carbons were observed in the HSQC–NOESY spectrum (Fig. 5): **A** H-1/**D** C-3, **D** H-1/**C** C-3, **C** H-1/**B** C-2, **B** H-1/**A** C-3, **E** H-1/**D** C-2, **F** H-1/**E** C-2, **G** H-1/**F** C-4, and **H** H-1/**G** C-3. Hence, K71 includes branched octasaccharide K units with one GlcNAc and three Rha residues in the main chain and one GlcA and three Rha residues in the side chain (Fig. 3A).

This structure was confirmed by Smith degradation of the CPS, which resulted in an oligosaccharide (OS) (Fig. 3A) as a result of cleavage of GlcA and four of the Rha residues. The OS contained two Rha residues, one GlcNAc residue, and a glyceraldehyde aglycon derived from a 2-substituted Rha residue. The structure of the OS was established by NMR spectroscopy as described for the CPS (for the assigned ^1^Н and ^13^C NMR chemical shifts, see Table 1). The OS structure was confirmed by determination of its molecular mass by negative ion mode electrospray ionization mass spectrum, which showed peaks of the [M + Na]^+^ ion at *m*/*z* 626.2263 and [M + K]^+^ ion at *m*/*z* 642.2017 against the calculated values *m*/*z* 626.2267 and 642.2006, respectively.

### Configuration of the K71 unit and assignment of the Wzy_KL71_ polymerase

As the K71 octasaccharide unit includes a single d-Glc*p*NAc, this residue was assigned as the first sugar due to the presence of *itrA3* in KL71 that codes for an ItrA3 d-Glc*p*NAc-1P initiating transferase. Hence, in the K71 CPS, there is a tetrasaccharide main chain of →3)-α-l-Rha*p*-(1→3)-α-l-Rha*p*-(1→2)−α-l-Rha*p*-(1→3)-β-d-Glc*p*NAc-(1→, and an α-l-Rha*p*-(1→3)-α-l-Rha*p*-(1→4)-β-d-Glc*p*A-(1→2)-l-Rha*p* side branch joined α-(1→4) to the terminal l-Rha*p* residue in the main chain (Fig. 3A). The bond between individual K units is therefore a β-d-Glc*p*NAc-(1→3)-α-l-Rha*p* linkage. As the bond between K units is the only linkage that differs between K71 and K74, which has a six sugar main chain and a disaccharide side chain, and the linkages formed by the encoded glycosyltransferases were previously assigned for K74 [as shown in Fig. 3B (6)], the Wzy_KL71_ polymerase encoded by KL71 could be assigned to the β-d-Glc*p*NAc-(1→3)-α-l-Rha*p* linkage. This assignment is supported by Wzy_KL71_ sharing 48.73% amino acid sequence identity with Wzy_KL55_ reported to form a β-d-Glc*p*NAc-(1→3)-α-l-Rha*p* linkage between units in the *A. baumannii* K55 CPS (6) (see Fig. 3).

### Distribution of KL71 and KL74 in *A. baumannii* genomes

A total of 27,079 *A*. *baumannii* genome assemblies available in NCBI (as of 21 May 2024) were screened for the presence of the KL71 CPS biosynthesis gene cluster to assess the distribution of the K71 CPS in *A. baumannii*. The KL71 sequence was found in only two further genomes from isolates PUMA0075 and IK20 (NCBI assembly accession numbers GCA_030552105 and GCA_032941345, respectively). Both are environmental isolates recovered in Southeast Asia and belong to ST142. Similarly, KL74 was only otherwise identified in two genomes that also belong to ST142. These are isolate A156 (NCBI assembly accession number GCA_022937765) from a respiratory specimen recovered in China in 2018 and isolate ARLG-1813 (NCBI assembly accession number GCA_002136815) recovered in the United States of America. The average nucleotide identity of these six genomes was >99.8%, indicating that they are highly related. This suggests that both KL71 and KL74 are rarely encountered CPS types in *A. baumannii* and are currently restricted to the ST142 lineage.

## DISCUSSION

The majority of extensively resistant *A. baumannii* that warrant alternate therapeutic inventions belong to the globally disseminated clonal complex, CC2. ST142 is a relatively uncommon lineage with only four ST142 isolates reported in the literature to date. These include one isolate recovered from the cerebrospinal fluid of a hospitalized patient in China (20); an environmental isolate recovered from tank milk in Bogor, Indonesia (described above) (21); and BAL_309 and 48_n isolates recovered from the same ICU at the HTD in Ho Chi Minh City, Vietnam (4). In this study, we report that isolate 48_n is extensively antibiotic resistant and carries a large suite of acquired resistance genes that account for the observed resistance profile. However, while BAL_309 was recovered during an outbreak of VAP in the ICU, 7 years after 48_n, this isolate is susceptible to antibiotics. The *oxa10* gene does not confer resistance to carbapenems in *Escherichia coli*, where it is usually studied, and its ability to confer resistance to some carbapenem antibiotics in *A. baumannii* (8) is not widely appreciated. Here, the carbapenem resistance phenotype of 48_n, namely, susceptible to imipenem but resistant to meropenem and doripenem, is consistent with the phenotype reported earlier (8) for *oxa10* in *A. baumannii*.

Structural resolution of the CPS produced by 48_n confirmed that the sequence of the K71 unit is identical to that of K74 produced by BAL_309 (6) and that the two CPSs differ only in the nature of the linkage between K units that is formed by the Wzy polymerase. This difference arises due to the replacement of the *wzy* gene at the K locus, for which the encoded products share little sequence homology and select different donor sugars to participate in the bond (l-Rha*p* for Wzy_KL71_ and d-Glc*p*A for Wzy_KL74_), resulting in a change to the overall topology where different numbers of monosaccharides are present in the main chain and side branch of the CPS (Fig. 3B; K71 = four main-chain and four side-branch sugars; K74 = six main-chain and two side-branch sugars).

These two structures also share the same monosaccharide constituents as five other CPS types previously described for *A. baumannii* (Fig. 3). These types (K55, K85, K86, K144, and K239) are all initiated by d-Glc*p*NAc followed by an l-Rha*p* sugar α-(1→3) linked to this residue by a Gtr82 glycosyltransferase. This group of related CPS can be further split into two subgroups based on the sequence of the 2-substitued and 3-substituted Rha residues in the main chain, and this is determined by the activity of either the multifunctional Gtr112 glycosyltransferase that forms three linkages (Fig. 3B) or bifunctional Gtr81 glycosyltransferase that forms two linkages (Fig. 3C) as described previously (6). Consistent with this, the corresponding gene clusters for these CPSs share a central *ugd4-rmlBDAC* gene module (Fig. 1B) but differ in the number and types of glycosyltransferase genes and/or the sequence for the Wzy polymerase. While four of these CPSs share a β-d-Glc*p*NAc-(1→3)-α-l-Rha*p* linkage between their K units, the encoded Wzy polymerases share little sequence identity to each other despite belonging to the same protein family (Table 2). Hence, the specificity of Wzy polymerases is complex and likely involves multiple structural epitopes present in the *A. baumannii* CPS.

**TABLE 2:** Comparison of Wzy polymerases showing amino acid sequence identity matrix on the right

Wzy	Protein family	Length (aa)	No. of TMS	Unit topology^*a*^	Linkage	Wzy_KL71_	Wzy_KL55_	Wzy_KL144_	Wzy_KL86_	Wzy_KL239_	Wzy_KL74/_ Wzy_KL85_
Wzy_KL71_	EpsG	359	9	4 + 4	β-d-Glc*p*NAc-(1→3)-l-Rha*p*	100	48.73	23.91	21.17	22.46	19.62
Wzy_KL55_	EpsG	365	10	4 + 4	β-d-Glc*p*NAc-(1→3)-l-Rha*p*	48.73	100	19.32	19.47	22.46	17.57
Wzy_KL144_	EpsG	321	9	6 + 0	β-d-Glc*p*NAc-(1→3)-l-Rha*p*	23.91	19.32	100	27.56	20.00	23.21
Wzy_KL86_	EpsG	327	9	4 + 3	β-d-Glc*p*NAc-(1→3)-l-Rha*p*	21.17	19.47	27.56	100	16.91	19.80
Wzy_KL239_	–^*b*^	366	9	4 + 3	β-d-Glc*p*NAc-(1→2)-l-Rha*p*	22.46	22.46	20.00	16.91	100	19.92
Wzy_KL74/KL85_^*c*^^*c*^	EpsG	412	9	6 + 2/1^*d*^	β-d-Glc*p*NAc-(1→3)-d-Glc*p*A	19.62	17.57	23.21	19.80	19.92	100

^*a*^ Number of residues in the K-unit main chain versus number of residues in the K-unit side branch.

^*b*^ No detected Pfam.

^*c*^ Wzy_KL74_ from KL74 and KL85 are 98.8% identical.

^*d*^ K74 has a disaccharide side branch, whereas K85 has a monosaccharide side branch.

^*e*^ TMS = transmembrane segments.

## MATERIALS AND METHODS

### Isolate, cultivation, and resistance profiling

*A. baumannii* isolate 48_n was obtained in 2004 from a patient nasal carriage sample at the HTD in Ho Chi Minh City, Vietnam (4). The antibiotic resistance profile of 48_n was determined as described previously (22). For CPS structural analysis, cells of 48_n were cultivated in 2TY medium for 16 h, harvested by centrifugation (10,000 × *g*, 20 min), washed with phosphate buffered saline, then suspended in an acetone–water mixture [7:3 (vol/vol)], precipitated by centrifugation, and dried on air.

### Whole-genome assembly and bioinformatics analyses

Short read data for *A. baumannii* 48_n (SRA accession number SRR26815235) was quality checked then *de novo* assembled using SPAdes v.3.15.5 (23). The assembled genome consisted of 277 contigs with a total length of 4,182,363 bases, an N50 of 60.7 kb, and a read depth of 80×. The assembly was annotated using Prokka (https://github.com/tseemann/prokka) and uploaded to NCBI under accession number JAWXYJ000000000 (BioProject number PRJNA893697 and BioSample number SAMN38236054). OrthoANI values were calculated with the OAT tool (24).

Multilocus sequence typing was performed using the *A. baumannii* IP and OX schemes available at https://pubmlst.org/bigsdb?db=pubmlst_abaumannii_seqdef. Resistance genes were identified using NCBI AMRFinder (https://www.ncbi.nlm.nih.gov/pathogens/antimicrobial-resistance/AMRFinder/). Kaptive v.2.0.7 (https://github.com/klebgenomics/Kaptive) was used to identify the sequence at the K locus using the latest *A. baumannii* KL reference sequence database that includes 241 KL (14). The KL71 sequence in the *A. baumannii* 48_n genome was extracted, manually annotated according to the established nomenclature system (9), and submitted to NCBI GenBank under accession number PP621479.1.

### Isolation of CPS

Cell mass (3.05 g) was extracted with 45% aqueous phenol (70°C, 1 h) (15). The extract was dialyzed without layer separation and freed from insoluble contaminations by centrifugation. The resultant solution was concentrated and treated with cold aqueous 50% CCl_3_CO_2_H at 0°C for 1 h; after centrifugation, the supernatant was dialyzed against distilled water. A crude CPS sample (100 mg) was hydrolyzed with 2% CH_3_CO_2_H (100°C, 2 h). Fractionation of the products by gel-permeation chromatography on a column (56 × 2.5 cm) of Sephadex G-50 Superfine (Healthcare) in 0.05-M pyridinium acetate (pH 4.5) as eluent gave a purified CPS sample (56 mg).

### Chemical analyses

A CPS sample (1 mg) was hydrolyzed with 2-M CF_3_CO_2_H (120°C, 2 h) then blown in air current with MeOH. The products were reduced with NaBH_4_ in 1-M NH_4_OH (0.5 mL, 10 mg, 20°C, 1 h) and after blowing off in AcOH 2% in MeOH acetylated by a 1:1 (vol/vol) mixture of pyridine and Ac_2_O (120°C, 2 h). The alditol acetates obtained were analyzed by GLC on a Maestro chromatograph (Agilent 7820; Interlab, Russia) equipped with an HP-5 column (0.32 mm × 30 m) using a temperature program of 160°C (1 min) to 290°C at 7 °C/min.

To determine the absolute configuration of the monosaccharides, a CPS sample (1 mg) was hydrolyzed with 2-M CF_3_CO_2_H (120°C, 2 h) then blown in air current with MeOH. Following this, (+)−2-octanol (0.1 mL) and CF_3_CO_2_H (15 µL) were added (16 h, 120°C.) After blowing off with MeOH, the products were acetylated with a 1:1 (vol/vol) mixture of pyridine and Ac_2_O (120°C, 2 h). The acetylated 2-octyl glycosides were analyzed by GLC as indicated above.

### Smith degradation

A CPS sample (21 mg) was oxidized with aqueous NaIO_4_ (25 mg in 3.2 mL of H_2_O) at 20°C for 40 h in the dark and reduced with NaBH_4_ (25 mg) at 20°C for 16 h. The excess of NaBH_4_ was destroyed with concentrated HOAc; the solution was evaporated; then methanol was added to the residue (3 × 1 mL) and evaporated. The residue was dissolved in 0.3-mL water and applied to a column (80 × 1.6 cm) of TSK HW-40 (S) in 1% HOAc. A modified polysaccharide was eluted with aqueous 0.1% HOAc and hydrolyzed with 2% CF_3_CO_2_H (100°C, 2 h). Fractionation of the products by gel-permeation chromatography on a column (80 × 1.6 cm) of TSK HW-40 (S) in 1% HOAc gave an OS sample (7.4 mg).

### NMR spectroscopy

Samples were deuterium-exchanged by freeze-drying from 99.9% D_2_O and then examined as solutions in 99.95% D_2_O. NMR spectra were recorded on a Bruker Avance II 600 MHz spectrometer (Germany) at 60°C. Sodium 3-trimethylsilylpropanoate-2,2,3,3-d_4_ (δ_H_ 0, δ_C_ −1.6) was used as internal reference for calibration. Two-dimensional NMR spectra were obtained using standard Bruker software, and Bruker TopSpin v.2.1 program was used to acquire and process the NMR data. A 60-ms MLEV-17 mixing time and a 200-ms spin-lock time were used in ^1^H,^1^H TOCSY and ROESY experiments, respectively. A 60-ms delay was used for evolution of long-range couplings to optimize a ^1^H,^13^C HMBC experiment for the coupling constant of *J*_H,C_ 8 Hz. A mixing time of 0.3 s was used in a HSQC–NOESY experiment.

### Mass spectrometry

High-resolution electrospray ionization mass spectrometry was performed in the negative ion mode using a micrOTOF II instrument (Bruker Daltonics). An oligosaccharide sample (~50 ng/L) was dissolved in a 1:1 (vol/vol) water–acetonitrile mixture and injected with a syringe at a flow rate of 3 µL/min. Capillary entrance voltage was set at 3,200 V, and the interface temperature was set at 180°C. Nitrogen was used as the drying gas. Mass range was from *m*/*z* 50 to 3,500. Internal calibration was done with ESI Calibrant Solution (Agilent).

## Note

TMS = transmembrane segment
